# Dataset on the fauna and biology of flesh flies (Diptera: Sarcophagidae) in the region of European Russia

**DOI:** 10.3389/finsc.2026.1670763

**Published:** 2026-01-23

**Authors:** Stjepan Krčmar, Alexander B. Ruchin, Mikhail N. Esin, Irina G. Esina, Anatoliy A. Khapugin

**Affiliations:** 1 Department of Biology, Josip Juraj Strossmayer University of Osijek, Osijek, Croatia; 2 Joint Directorate of the Mordovia State Nature Reserve and «Smolny» National Park, Saransk, Russia; 3 Institute of Environmental and Agricultural Biology (X-BIO), Tyumen State University, Tyumen, Russia

**Keywords:** diptera, diversity, flesh flies, Mordovia, Russia, Sarcophagidae, traps

## Abstract

The dataset presents results from studies of Sarcophagidae conducted in 2008, 2013, 2014, and 2016–2024 in the central part of European Russia (Republic of Mordovia). A total of 2,162 males representing 58 species and 10 genera were reliably identified. The highest species diversity of Sarcophagidae was recorded in the Mordovia State Nature Reserve. For the first time in Russia, a new species — *Sarcophaga* (*Heteronychia*) *slovaca* (Povolný and Slamečková, 1967) — was documented. The highest species diversity of Sarcophagidae was recorded in forest glades and floodplains (39 species each). *Sarcophaga* (*Sarcophaga*) *variegata* (Scopoli, 1763) reached its highest abundance in meadows and was relatively common along forest edges. *Sarcophaga* (*Sarcotachinella*) *sinuata* Meigen, 1826 and *Sarcophaga* (*Mimarhopocnemis*) *granulata* Kramer, 1908 predominated in deciduous forests. *Metopia grandii* Venturi, 1953 and *Metopia argyrocephala* (Meigen, 1824) were most abundant in burned forest areas. In terms of collection methods, the greatest number of both specimens and species was obtained using yellow pan traps and Malaise traps.

## 1 Introduction

In recent years, researchers have identified a global decline in insect populations and taxonomic diversity, both worldwide and within specific macro-regions (1, 2). A wide range of contributing factors has been identified, including global climate change, urbanization, habitat degradation, megafires, and ecosystem pollution. These processes trigger a cascade of secondary effects that also negatively impact insect population dynamics (3–6).

Insects play a vital role in terrestrial ecosystems. They are integral to all trophic chains without exception, and the normal functioning of ecosystems is difficult to imagine in their absence (7). However, numerous studies have shown that our knowledge of insects remains limited and often vague, particularly concerning local and regional faunas, especially the insect fauna of remote or hard-to-access areas and that of protected areas (8, 9). Furthermore, many aspects of insect distribution and species biology remain poorly understood. This lack of knowledge ultimately affects the development of effective species conservation programs, long-term ecosystem management, and the provision of ecosystem services (10, 11).

Due to the relatively good preservation of many ecosystems and the diversity of landscapes, Russia plays an important role in species conservation. Nonetheless, knowledge of the regional insect fauna and species biology is often superficial and based on century-old literature (10). At that time, Rohdendorf (1937) compiled a list of flesh flies (Sarcophagidae) of the USSR, richly illustrated and including keys for nearly every Palaearctic species then known (12). Recently, the insect fauna of Russia has been studied quite intensively, and many new records have been described (13–16), as well as new species (17–20). Flesh flies (Sarcophagidae) is a large family comprising over 2,800 described species distributed worldwide, which are divided into three subfamilies: Miltogramminae, Paramacronychiinae, and Sarcophaginae (21). At present, about 310 species of flesh flies occur in Europe (22, 23), while more than 150 species are known from Central Europe (24). Most members of this family are parasitoids or predators, with a relatively small number of species specializing in feeding on vertebrate carrion (25, 26). However, the dietary habits of Sarcophagidae are not limited to carrion. Some species are coprophagous, others inhabit nests of termites and ants, certain species cause myiasis in mammals and amphibians, and others prey on arachnid eggs, lepidopteran larvae, or bee pupae. Some members of the family are even highly specialized parasitoids of other arthropods (26–28).

The aim of our study is to analyze a dataset containing information on the fauna and biology of flesh flies (Diptera: Sarcophagidae) in one of the most thoroughly studied regions of central European Russia, the Republic of Mordovia. The objectives of the study were as follows:

1. to determine the number of Sarcophagidae species present in the fauna of the Republic of Mordovia.
2. to assess species diversity within the two largest protected areas–the Mordovia State Nature Reserve (MSNR) and Smolny National Park (SNP).
3. to investigate the abundance of recorded species.

## 2 Materials and methods

### 2.1 Study area

The Republic of Mordovia is located within the Volga Upland and the Oka–Don Lowland. The region is characterized by hilly terrain with elevations ranging from 200 to 300 meters above sea level, while the Oka–Don Lowland has a more level topography. The republic is situated between 42°11′ and 46°45′ E longitude and 53°38′ and 55°11′ N latitude. Its maximum extent is 298 km from west to east and 140 km from north to south, covering an area of 26,121 km². The territory lies within the temperate climatic zone, with clearly defined seasonal changes. Positioned in a moderately continental climate sector, the region experiences fluctuating precipitation patterns, alternating between wet and dry years. The soil composition includes a mix of sod-podzolic soils, gray forest soils, and chernozems. Natural vegetation is dominated by pine forests mixed with spruce, oak groves, and meadow steppes. The prevalence of forest-steppe landscapes supports agricultural development, and many landscapes have been altered by human activity.

The Mordovia State Nature Reserve was established in 1936 in the Temnikov District (northwestern part of the republic, within the Oka–Don Lowland). It covers an area of 321 km² and preserves natural landscapes of pine and broad-leaved forests typical of the glacial outwash plains of the forest-steppe zone. Smolny National Park is located in the Ichalki and Bolshoe Ignatovo Districts (within the Volga Upland) and was founded in 1995. It occupies an area of 365 km².

### 2.2 Sampling methods

Field research was conducted in 2008, 2013, 2014, and from 2016 to 2024. To collect specimens, all standard entomological sampling methods were employed, including hand-held sweep nets, pitfall traps, beer traps, yellow pan traps, and Malaise traps (29).

For the yellow pan traps, yellow plastic dishes with a diameter of 20 cm and a capacity of 1 liter were used. Each dish was filled with up to 500 ml of water mixed with a small amount of detergent. These traps were typically deployed in a single line of 10 traps placed in grassy vegetation in meadows, forest glades, and forest edges. The distance between individual dishes in a line varied from 1 to 3 meters. The exposure time for a single line of traps at a given locality ranged from 3 to 7 days, depending on the region. The Malaise traps used were homemade and modeled after the Townes-style Malaise trap (30), constructed with wooden frames and covered in white fabric. Collection containers were filled with 70% ethanol. These traps were placed at forest edges, usually with one trap installed per habitat for the entire season. Trap maintenance and sample collection were carried out at intervals ranging from 3 to 12 days. Pitfall traps were arranged in single lines of 10 traps each. Each trap consisted of a 0.5-liter plastic cup filled with 150 ml of a 4% formalin solution as a preservative. These traps were installed across a variety of habitats, including meadows, fields, shelterbelts, and forests. The distance between traps in a line ranged from 1.5 to 2 meters. Typically, pitfall traps remained in place for 10 to 28 days. Beer traps were constructed from 1.5-liter and 5-liter plastic containers with openings cut into the sides to allow insect entry. These traps were hung on tree branches in forests and forest edges at heights ranging from 1.5 to 12 meters above ground level. The bait consisted of fermented beer mixed with sugar. Exposure periods for beer traps ranged from 7 to 15 days. All sampling methods were applied from April to November, coinciding with the period of peak insect activity in central European Russia. The collected specimens of flesh flies were stored and preserved in a 96% ethanol solution.

During specimen collection, researchers recorded habitat type (across 12 distinct habitat categories – meadow, edge of pine forest, deciduous forest, burned area of the forest, forest glade, floodplain, pine forest, steppificated site, sandy area, mixed forest, quarry, forest belt along agrofields), dates, geographic coordinates, and original locality names. This information was also included in the dataset. In total, data from 86 localities were used (31).

### 2.3 Taxonomic analysis

All collected specimens were examined, and most of them were identified in the laboratory of the Department of Biology, Josip Juraj Strossmayer University of Osijek, Croatia. Identifications were carried out according to the following keys for Sarcophagidae (24, 32–34) and descriptions and illustrations (35–40). The nomenclature and classification follow the Catalogue of Sarcophagidae of the World and the Fauna Europaea database (21, 22, 41). Only male specimens were used for analysis. Species concepts within the Sarcophagidae traditionally pay most attention to males. Males are generally better represented in collections, probably because they expose themselves more readily to collectors through hill-topping behavior and territoriality (33). Males are almost always dominant, usually forming at least 80% of the catch (34). Male terminalia have long been recognized as providing the most reliable structures for species identification (33). Namely, the copulatory apparatus of male specimens is highly specific at the species level, enabling easy identification by direct comparison with figures in identification keys (32). When identifying females, many pitfalls exist that may lead to incorrect results; therefore, females caught in copula should always be pinned with their males, as they are valuable reference specimens (32). Male terminalia were dissected and partially prepared for identification according to the method described by Richet et al. (34). Male abdomens were dissected and soaked in a 10% KOH solution for 72 hours. After that, male abdomens were immersed in a 96% ethanol solution for one hour. The phallus, pregonites and postgonites, sternite 5, cerci, and surstyli were separated from the abdomen and placed into 2 ml plastic tubes. The plastic tubes were filled with a 96% ethanol solution. Identification of flesh flies was performed using a Carl Zeiss Jena stereomicroscope with 40× magnification. All identified specimens have the following information recorded: locality and date of collection, collector(s), number and sex of specimens, sampling method, and geographical coordinates.

### 2.4 Statistical analysis

Ordination was performed using PCA (principal component analysis). All statistical analyses have been performed using PAST 4.11 software. Using this technique, we identified major gradients in the spatial arrangement of the studied species selected for analysis. Species abundance was used as the response variable in the PCA. For interpretation of the ordination axes, groups of the studied habitats were plotted onto the PCA ordination diagram as supplementary environmental data. Statistical analysis was carried out using standard Microsoft Excel software. To assess species diversity, abundance, and dominance, the following widely used ecological indices were applied: Shannon Index, Simpson Index, Margalef Index, Pielou Index, and Berger–Parker Index.

## 3 Results

Data from the dataset can be uploaded as a single XLSX file to GBIF (https://www.gbif.org/dataset/c4273248-a152-40c7-aabc-a1d205fcdfe7). It contains 770 rows, and each row represents a set of data. The columns contained in it are as follows (**Table 1**).

**Table 1:** Description of the data in the dataset.

Column label	Column description
occurrenceID	An identifier for the occurrence (as opposed to a particular digital record of the occurrence)
basisOfRecord	The specific nature of the data record: Human Observation
eventDate	The date when material from the trap was collected or the range of dates during which the trap collected material
scientificNam	The full scientific name including the genus name and the lowest level of taxonomic rank with the authority
kingdom	The full scientific name of the kingdom in which the taxon is classified
decimalLatitude	The geographic latitude of location in decimal degrees
decimalLongitude	The geographic longitude of location in decimal degrees
sex	The sex of the biological individual(s) represented in the occurrence
country	The name of the country in which the location occurs
countryCode	The standard code for the country in which the location occurs
individualCount	The number of individuals represented present at the time of the occurrence
year	The integer year in which the event occurred
month	The ordinal month in which the event occurred
day	The integer day of the month on which the event occurred
recordedBy	A person, group, or organization responsible for recording the original occurrence
identifiedBy	A list of names of people who assigned the taxon to the subject
habitat	A category or description of the habitat in which the Event occurred
samplingProtocol	The names of the methods or protocols used during an event
locality_original	The specific description of the place. This term may contain information modified from the original to correct perceived errors or standardize the description
georeferenceSources	A list of maps, gazetteers, or other resources used to georeference the Location
coordinateUncertaintyInMeters	The maximum uncertainty distance in metres
geodeticDatum	The ellipsoid, geodetic datum, or spatial reference system (SRS) upon which the geographic coordinates given in decimalLatitude and decimalLongitude is based

We reliably identified a total of 2,162 male specimens of Sarcophagidae, representing 58 species and 10 genera (**Table 2**). The most widespread genus was *Sarcophaga*, which accounted for 39 species (67.2%) and 1,652 specimens (76.4%) (**Table 2**).

**Table 2:** Diversity and abundance of Sarcophagidae species in the Republic of Mordovia (dataset).

Species	MSNR	SNP	Other district	Total number of specimens
*Amobia signata* (Meigen, 1824)	3			3
*Angiometopa falleni* Pape, 1986	33			33
*Blaesoxipha* (*Servaisia*) *erythrura* (Meigen, 1826)	4		1	5
*Blaesoxipha* (*Tephromyia*) *grisea* (Meigen, 1826)	4	5	1	10
*Blaesoxipha* (*Blaesoxipha*) *laticornis* (Meigen, 1826)	2			2
*Blaesoxipha* (*Blaesoxipha*) *plumicornis* (Zetterstedt, 1859)	7	5	4	16
*Blaesoxipha* (*Servaisia*) *rossica* Villeneuve, 1912	33		12	45
*Blaesoxipha* (*Blaesoxipha*) *unicolor* (Villeneuve, 1912)	9			9
*Brachicoma devia* (Fallén, 1820)	14	75	23	112
*Metopia argyrocephala* (Meigen, 1824)	76		2	78
*Metopia campestris* (Fallén, 1810)	5	2		7
*Metopia grandii* Venturi, 1953	97	1	1	99
*Miltogramma villeneuvei* Verves, 1982			2	2
*Oebalia minuta* (Fallén, 1810)	1			1
*Ravinia pernix* (Harris, 1780)	22	4	15	41
*Sarcophaga* (*Helicophagella*) *agnata* (Rondani, 1860)		1		1
*Sarcophaga* (*Parasarcophaga*) *albiceps* Meigen, 1826	14		3	17
*Sarcophaga* (*Heteronychia*) *ancilla* Rondani, 1865			3	3
*Sarcophaga* (*Rosellea*) *aratrix* Pandellé, 1896	14	7	16	37
*Sarcophaga* (*Liopygia*) *argyrostoma* (Robineau-Desvoidy, 1830)			2	2
*Sarcophaga* (*Heteronychia*) *bulgarica* (Enderlein, 1936)	4			4
*Sarcophaga* (*Robineauella*) *caerulescens* Zetterstedt, 1838	21	4	9	34
*Sarcophaga* (*Sarcophaga*) *carnaria* (Linnaeus, 1758)	12	41	110	163
*Sarcophaga* (*Helicophagella*) *crassimargo* Pandellé, 1896		4	1	5
*Sarcophaga* (*Heteronychia*) *depressifrons* Zetterstedt, 1845	34	1	13	48
*Sarcophaga* (*Heteronychia*) *dissimilis* Meigen, 1826	6		3	9
*Sarcophaga* (*Liosarcophaga*) *emdeni* Rohdendorf, 1969	10	9		19
*Sarcophaga* (*Mimarhopocnemis*) *granulata* Kramer, 1908	4	2	105	111
*Sarcophaga* (*Heteronychia*) *haemorrhoa* Meigen, 1826	16	5	20	41
*Sarcophaga* (*Heteronychia*) *haemorrhoides* Böttcher, 1913	6	6	35	47
*Sarcophaga* (*Liosarcophaga*) *harpax* Pandellé, 1896	1		2	3
*Sarcophaga* (*Thyrsocnema*) *incisilobata* Pandellé, 1896	43	55	52	150
*Sarcophaga* (*Sarcophaga*) *lehmanni* Müller, 1922	60	25	53	138
*Sarcophaga* (*Helicophagella*) *melanura* Meigen, 1826	5	1	12	18
*Sarcophaga* (*Mehria*) *nemoralis* Kramer, 1908	21			21
*Sarcophaga* (*Liosarcophaga*) *portschinskyi* (Rohdendorf, 1937)	15		3	18
*Sarcophaga* (*Pandelleana*) *protuberans* Pandellé, 1896	8	10	1	19
*Sarcophaga* (*Heteronychia*) *proxima* Rondani, 1860	3	2	2	7
*Sarcophaga* (*Helicophagella*) *rosellei* Böttcher, 1912	2	3	3	8
*Sarcophaga* (*Heteronychia*) *schineri* Bezzi, 1891	4	4		8
*Sarcophaga* (*Kramerea*) *schuetzei* Kramer, 1909	2		2	4
*Sarcophaga* (*Mehria*) *sexpunctata* (Fabricius, 1794)		4	4	8
*Sarcophaga* (*Pandelleisca*) *similis* Meade, 1876	3	3	18	24
*Sarcophaga* (*Sarcotachinella*) *sinuata* Meigen, 1826	6	1	81	88
*Sarcophaga (*Heteronychia*) *slovaca* (Povolný and Slamečková, 1967)		2	2	4
*Sarcophaga* (*Myorhina*) *socrus* Rondani, 1860	40	20	35	95
*Sarcophaga* (*Myorhina*) *soror* Rondani, 1860			1	1
*Sarcophaga* (*Bellieriomima*) *subulata* Pandellé, 1896	7	3	4	14
*Sarcophaga* (*Sarcophaga*) *subvicina* Rohdendorf, 1937	4	2	11	17
*Sarcophaga* (*Liosarcophaga*) *tuberosa* Pandellé, 1896	17		6	23
*Sarcophaga* (*Heteronychia*) *vagans* Meigen, 1826	10	4	9	23
*Sarcophaga* (*Sarcophaga*) *variegata* (Scopoli, 1763)	130	75	163	368
*Sarcophaga* (*Heteronychia*) *vicina* Macquart, 1835	1			1
*Sarcophaga* (*Myorhina*) *villeneuvei* Böttcher, 1912	3		48	51
*Sarcophila latifrons* (Fallén, 1817)	2	2	13	17
*Senotainia albifrons* (Rondani, 1859)	21	4		25
*Senotainia conica* (Fallén, 1810)			1	1
*Taxigramma elengatulum* (Zetterstedt, 1844)	2		2	4
**Total specimens**	**861**	**392**	**909**	**2162**
**Total species**	**49**	**34**	**45**	**58**
**Total species, %**	**84.5**	**58.6**	**77.6**	**100**
**Shannon Index**	**3.21**	**2.64**	**2.90**	
**Simpson Index**	**0.93**	**0.89**	**0.92**	
**Margalef Index**	**7.25**	**5.69**	**6.45**	
**Pielou Index**	**0.82**	**0.74**	**0.76**	
**Berger-Parker Index**	**0.15**	**0.19**	**0.17**	

*new record for Russia, MSNR (Mordovia State Nature Reserve), SNP (Smolny National Park). In bold: Similarity indices and total number of species and specimens.

The greatest species diversity of Sarcophagidae was recorded in the Mordovia State Nature Reserve (MSNR), with 49 species identified. Other areas outside the protected zones also exhibited high species diversity. In contrast, Smolny National Park (SNP) showed a lower species richness, which corresponded to the comparatively small number of specimens collected there.

*Sarcophaga* (*Heteronychia*) *slovaca* (Povolný and Slamečková, 1967) was recorded for the first time in Russia. This species inhabits humid mountain forests and may descend to lower forest limits, although its biology remains unknown (24, 34). Records of this species in Europe are quite rare and have been reported only from four European countries (the Czech Republic, France, Slovakia, and Ukraine) (36). This is a medium-sized (6.5–9.0 mm) flesh fly. Male terminalia are characterized by the following features: setae on sternite 5 are thickened and shortened, with the median setae only slightly longer than the others and having a slightly hooked tip (36). The protandrial segment bears a round patch of microtrichosity near the posterior margin, although it may sometimes be weakly visible (36). The epandrium is black (36). The cercus has a distinct, rounded dorsal preapical hump, is more or less uniformly covered with short, sparse setae, and has a bluntly tapering tip with a concave dorsal surface (36). The pregonite bears short, sparse setulae almost reaching the tip; the tip is rounded and curved medioventrally (36). The distiphalus has a proximal part of the harpes protruding in lateral view, rounded and continuous with the distal part (36). The apical processes are flattened, tapering, and directed apicoventrally; the juxta is short, with somewhat sclerotized, narrow, spoon-like basal processes that are shorter than the median part of the juxta in lateral view. The median part of the juxta is narrow, with short apical processes and a slight dorsal concavity in lateral view (36). The lateral styli distinctly widen apically, and the vesica is subrectangular with a slight median crease and pointed corners (36).

**Figure 1:**
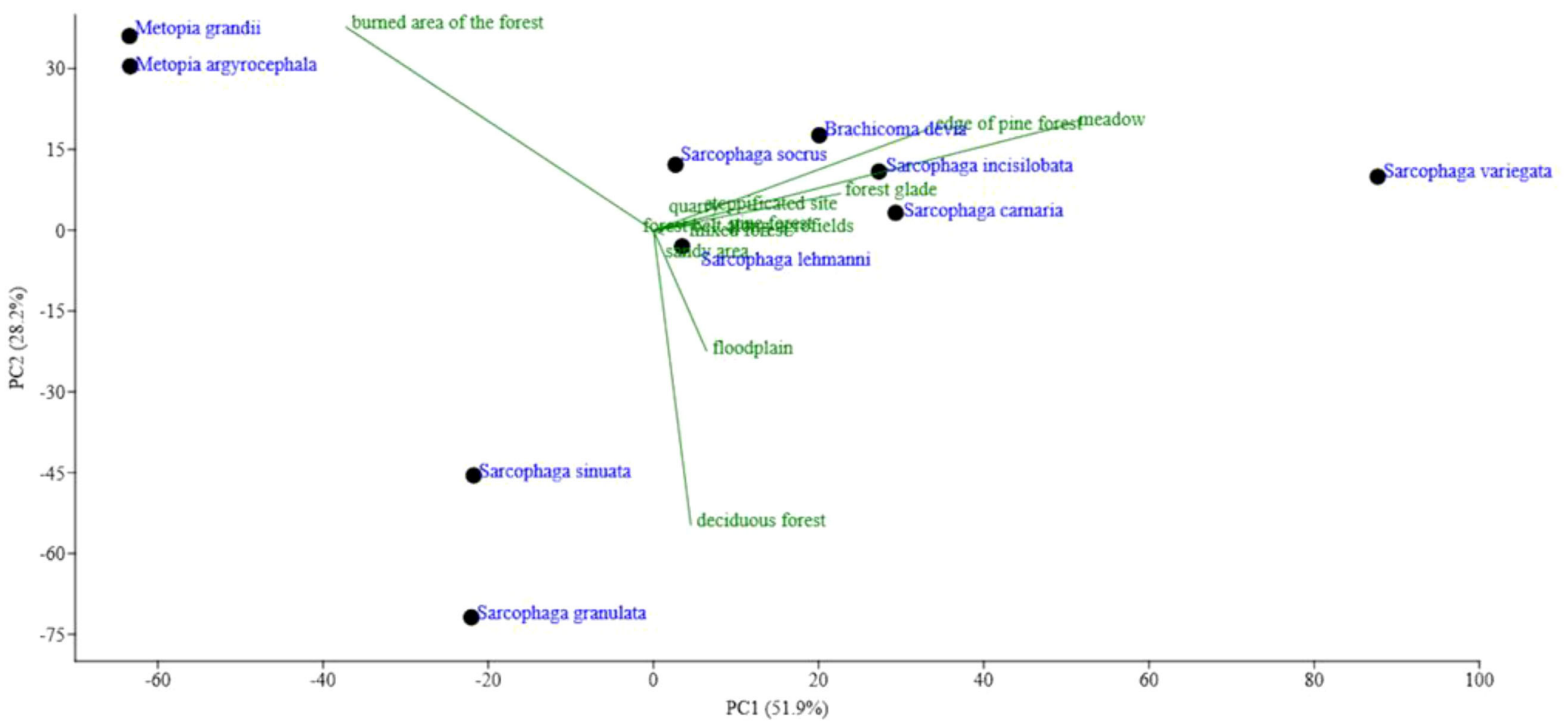
The diagram of the principal component analysis ordination of the selected species in the Republic of Mordovia (European Russia) based on the number of specimens collected at various habitats.

The highest abundance of specimens was recorded for the following ten species: *S.* (*Sarcophaga*) *variegata* (Scopoli, 1763), *S.* (*Sarcophaga*) *carnaria* (Linnaeus, 1758), *S*. (*Thyrsocnema*) *incisilobata* (Pandellé, 1886), *S.* (*Sarcophaga*) *lehmanni* Mueller, 1922, *Brachicoma devia* (Fallén, 1820), *S.* (*Mimarhopocnemis*) *granulata* Kramer, 1908, *Metopia grandii* Venturi, 1953, *S.* (*Myorhina*) *socrus* Rondani, 1860, *S.* (*Sarcotachinella*) *sinuata* Meigen, 1826, and *Metopia argyrocephala* (Meigen, 1824). Together, these species accounted for 64.8% of the total specimen count in the dataset. To investigate the habitat preferences of these species, a principal component analysis (PCA) was performed (**Figure 1**).

It was found that the majority of species clustered within a small area of the PCA diagram, indicating that they do not exhibit pronounced habitat preferences. Conversely, five species were positioned farther from this cluster. As shown in **Figure 1**, *S*. (*Sarcophaga variegata*) was notably distant from the other species, with its highest abundance recorded in meadows and a relatively frequent presence at forest edges. Our findings align with biological descriptions from other studies, which characterize this species as commonly found in open habitats such as meadows, pastures, parks, roadsides, and forest edges. Adults feed on decaying carrion, fruits, and sugary excretions of aphids (42, 43).

*S.* (*Sarcotachinella*) *sinuata* and *S.* (*Mimarhopocnemis*) *granulata* predominated in abundance within deciduous forests. The former species is described as highly hygrophilous; adults feed on feces and other decaying organic matter. Its larvae parasitize insects (Orthoptera, Lepidoptera) and can also develop in the carcasses of small vertebrates (43). In the Republic of Mordovia, deciduous forests are often moist habitats located in river floodplains, making these results consistent with the known biology of the species.

*Metopia grandii* and *Metopia argyrocephala* exhibited high abundances in burned forest areas. Both species are generally reported to prefer moist habitats such as forest edges and meadows (44). However, in our study area, it is difficult to classify burned areas as moist habitats. On the other hand, these burned sites have transformed into open meadow ecosystems characterized by considerable plant diversity (45). Given that *Metopia grandii* and *Metopia argyrocephala* feed on flowers, their high abundance in these habitats is more understandable.

**Figure 2:**
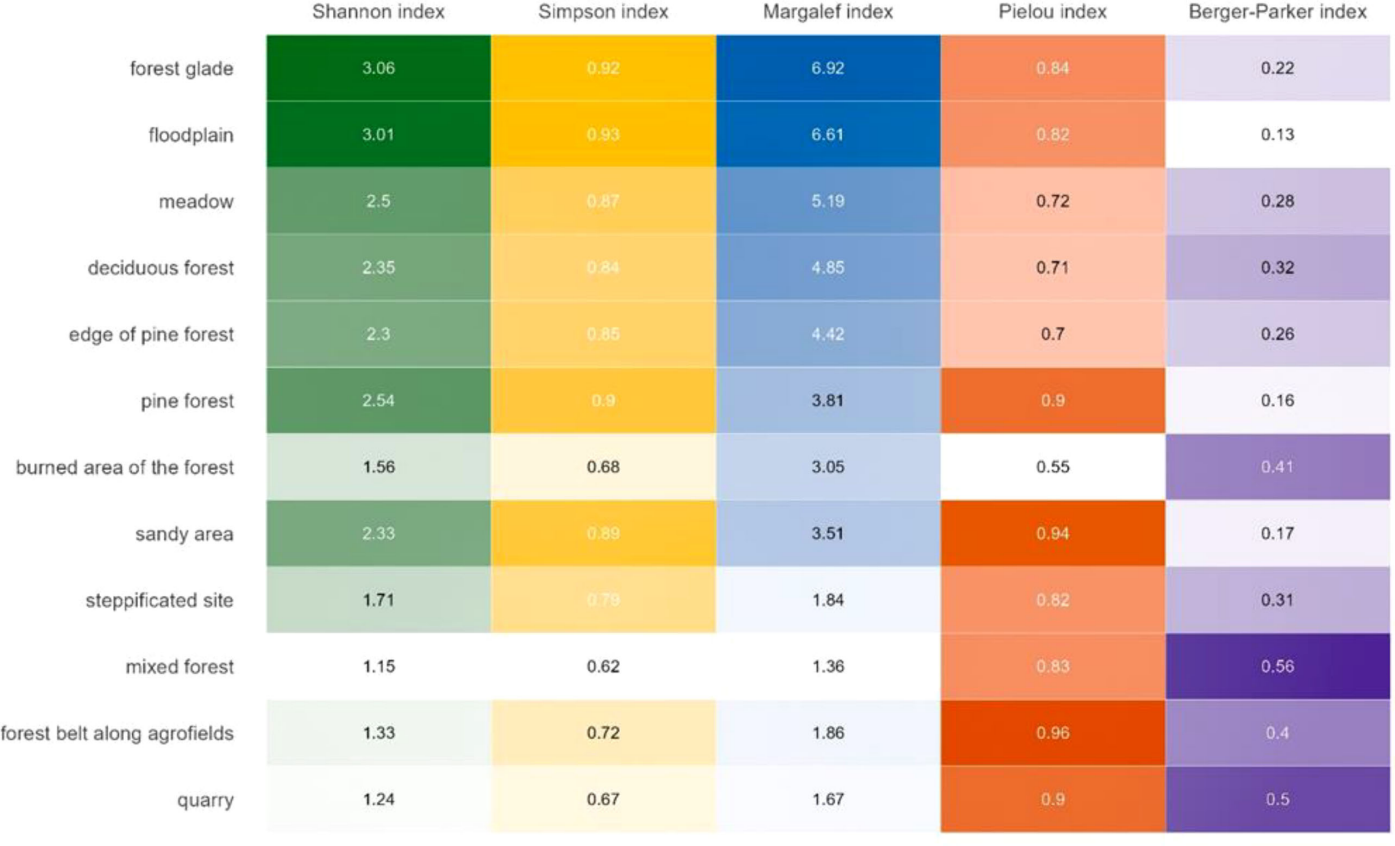
Indices of Sarcophagidae diversity in different habitats. The more intense the color, the higher the index value.

The greatest species diversity was recorded in forest glades and floodplains, with 39 species each. These habitats also showed the highest values of the Shannon and Margalef diversity indices, with no single species dominating (**Figure 2**). Although Sarcophagidae abundance was highest in meadows, species diversity was lower, with 32 species recorded and dominance by several species: *S*. (*Sarcophaga*) *variegata*, S. (*Sarcophaga carnaria*) and *S.* (*Thyrsocnema*) *incisilobata*.

Interestingly, burned forest areas exhibited high Sarcophagidae abundance despite relatively low species diversity. In this habitat, two species—*Metopia grandii* and *Metopia argyrocephala*—were essentially dominant (**Figure 1**).

**Figure 3:**
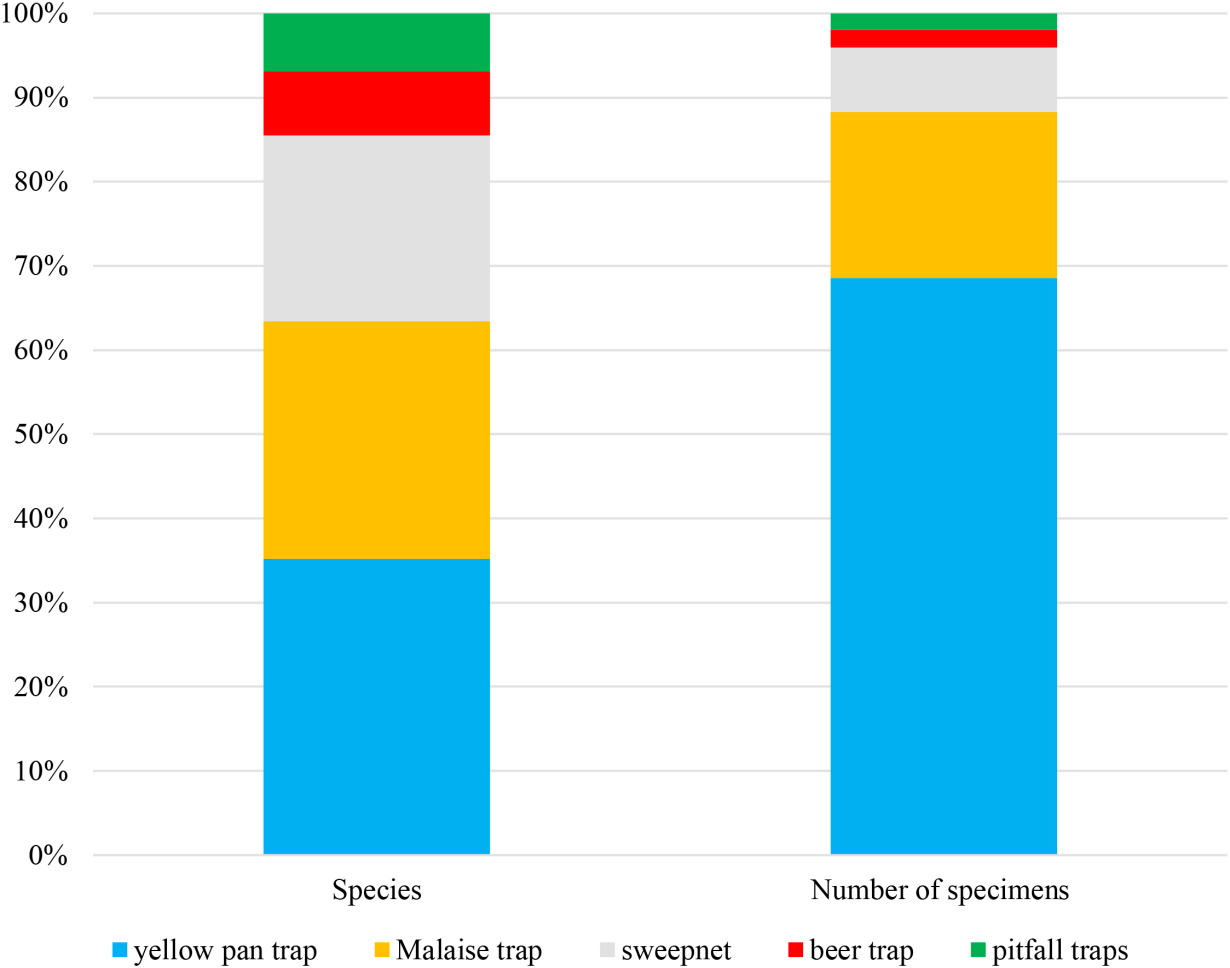
Number of species and specimens of Sarcophagidae collected using different sampling methods.

Regarding trapping methods, the greatest number of Sarcophagidae specimens and species were captured using yellow pan traps (**Figure 3**), followed by Malaise traps.

## 4 Discussion

### 4.1 Comparison with European Sarcophagidae fauna

A total of 58 flesh flies (Sarcophagidae) were recorded in the studied area, primarily due to continuous sampling from 2016 to 2024. The break in sampling in the previous period was caused by the sampling of other groups of insects and the processing and identification of these insects.

These 58 recorded species represent an incentive for further systematic research into the flesh fly fauna, as a much larger number of species have been recorded in neighboring and other European countries. The largest number of flesh fly species is known from Ukraine (179 species) (46, 47), followed by Croatia with 156 species (48, 49), Germany with 155 species (50), Hungary with 154 species (51), Turkey with 153 species (52), the Czech Republic with 143 species, Slovakia with 131 species (53), and Poland with 127 species (54). It is obvious that these differences in the number of recorded species in the immediate vicinity reflect differences in the intensity of faunal surveys conducted rather than differences in species composition. The genus *Sarcophaga* was the most abundant in recorded species, with 39 species recorded in this genus (**Table 2**). This is not surprising, as most species of the family Sarcophagidae belong to the subfamily Sarcophaginae, which comprises 323 genera and 2,028 species (55). The next most diverse genus was *Blaesoxipha*, with six species, of which only two were recorded in Smolny National Park, whereas all six species were present in the Mordovia State Nature Reserve. The genus *Blaesoxipha* is of particular biological interest, as it contains the majority of obligate insect parasites within the family Sarcophagidae (33). Hosts are mainly acridid grasshoppers and darkling beetles, although many other insect taxa may also serve as hosts (33). This was followed by the genus *Metopia*, with three species, while the remaining seven genera were represented by two or one species each.

### 4.2 Faunal similarities and differences

Five species - *Miltogramma villeneuvei, S. (Heteronychia) ancilla, S. (Myorhina) soror, Senotainia conica* and *S. (Liopygia) argyrostoma*—were collected exclusively outside the protected areas of the Mordovia State Nature Reserve (MSNR) and Smolny National Park (SNP). Almost the same number of species was recorded in MSNR (49) and in other districts outside protected areas (45), whereas the lowest number of species (34) was recorded in SNP (**Table 2**). The value of the Shannon index is quite high, ranging from 2.64 to 3.21 indicating a great diversity of flesh fly (Sarcophagidae) fauna, that is pointing to rich and diverse ecosystems (**Table 2**). The abundance is not overly dominated by any one species, since the abundance of the most abundant species *S*. (*Sarcophaga*) *variegata* ranges from 15.01% to 19.13% (**Table 2**).

### 4.3 Forensic significance of some species

Three species of forensic entomological significance were recorded in this study. Although the diversity of flesh flies visiting and colonizing large carrion in Central Europe is generally low (25), it is known that *S.* (*Liopygia*) *argyrostoma* colonizes decomposing human remains (56). In Switzerland, *S.* (*Liopygia*) *argyrostoma*, *S.* (*Pandelleisca*) *similis*, and *S.* (*Robineauella*) *caerulescens* have been recorded from human cadavers (57). Of these three species, *S.* (*Pandelleisca*) *similis* and *S.* (*Robineauella*) *caerulescens* were recorded in both protected areas (MSNR and SNP) (**Table 2**). Recently, the important role of *S.* (*Liopygia*) *argyrostoma* in accidental intestinal myiasis was confirmed (58).

### 4.4 Trap efficiency in Sarcophagidae sampling

In this study, yellow pan traps and Malaise traps were the most effective methods for sampling flesh flies (Sarcophagidae). Several studies have reported that Sarcophagidae are frequently and abundantly captured in pan traps, especially in open habitats (59, 60). Malaise traps are also widely used for sampling Sarcophagidae, particularly species belonging to the sarcosaprophagous ecological group (59, 61). Thus, the effective application of various sampling methods yielded excellent results, and the representativeness of the dataset appears to be very high.

### 4.5 Limitations of the study

The limitations of this study include the two important items:

a)pitfall trap and beer trap proved unsuitable for flesh flies collecting (**Figure 3**).

b)the large interruptions in flesh flies samplings from 2008 to 2013.

During the study, pitfall trap and beer trap have yielded subpar results compared to yellow pan trap and Malaise trap. There is a possibility that removing them from the study (after the first few years of results) and focusing on more successful traps would have resulted with larger numbers of collected specimens and species. Additionally, were it not for the breaks between 2008 and 2013, the samplings and results would reflect better on the overall state of flesh flies in studied areas, and would have provided more opportunities for assessing patterns, outliers and seasonality of flesh flies throughout years.

## 5 Conclusion

For the first time, verified records of 58 flesh flies (Sarcophagidae) species are reported from the Republic of Mordovia (central European Russia). The Sarcophagidae fauna of two large protected areas was studied in detail. An analysis was conducted on species occurrences based on captures using various trap types, and habitat preferences of the most abundant Sarcophagidae species were also examined. Dominant and mass-occurring Sarcophagidae species were identified. Further systematic entomological research, especially in the area of the Smolny National Park, will contribute to a better understanding of the flesh fly (Sarcophagidae) fauna as well as overall biodiversity of the Republic of Mordovia (Russia).

## Data Availability

The datasets presented in this study can be found in online repositories. The names of the repository/repositories and accession number(s) can be found in the article/supplementary material.
